# Knockdown *ATG5* gene by rAAV9 alleviates doxorubicin-induced cardiac toxicity by inhibiting GATA4 autophagic degradation

**DOI:** 10.3389/fphar.2024.1496380

**Published:** 2025-01-27

**Authors:** Ai-Li Xu, Zheng Shen, Shi-Hao Wang, Hai-Yun Luan, Yong Xu, Ze-Chun Kang, Zi-Qi Liao, Jie Liu, Xiao-Lei Duan, Wei-Hua Bian, Hui Sun, Xin Xie

**Affiliations:** ^1^ Department of Cardiology, The Binzhou Affiliated Hospital, Binzhou Medical University, Binzhou, China; ^2^ Department of Cardiology, Institute of Cardiovascular Diseases, First Affiliated Hospital of Dalian Medical University, Dalian, China

**Keywords:** Atg5, autophagy, cardiac toxicity, doxorubicin, oxidative stress

## Abstract

Doxorubicin (DOX) is a prevalent chemotherapeutic drug for treating several malignancies. However, the mechanisms of DOX induced cardiac toxicity is not fully understood. Previous studies have demonstrated that autophagy activation is essential in DOX-induced cardiac toxicity. Nevertheless, studies on the role of autophagy protein 5 (ATG5) in DOX-induced cardiac toxicity remain limited. Therefore, this study aimed to investigate the role of ATG5 in DOX-induced cardiac toxicity. Mice were intravenously administered DOX (5 mg/kg) for 4 weeks to establish a cardiac toxicity model. Heart function was determined using echocardiography, and cardiac tissue was assessed for protein expression, mRNA levels, fibrosis, and immunofluorescent staining. DOX treatment upregulated autophagy-related gene expression but inhibited autophagic flux *in vitro* and *in vivo*. DOX–treated mice exhibited decreased heart function and cardiomyocyte size and increased cardiac fibrosis, oxidative stress, and apoptosis. These effects of DOX were partially alleviated by rAAV9 expressing shRNA-ATG5 and deteriorated by rAAV9-ATG5. We demonstrated that genetic *ATG5* knockdown or autophagy inhibition by chemical inhibitors increased GATA4 protein expression, which was reduced by ATG5 overexpression or autophagy activator *in vitro* and *in vivo*, suggesting that ATG5-mediated autophagy promoted GATA4 degradation. Moreover, enforced GATA4 re-expression significantly counteracted the toxic effects of ATG5 on DOX-treated hearts. In conclusion, our study demonstrated that manipulating ATG5 expression to regulate GATA4 degradation in the heart may be a promising approach for DOX-induced cardiac toxicity.

## Introduction

Doxorubicin (DOX) remains a powerful first-line drug in clinical use for the treatment of several cancers, including leukemia, lymphoma, and breast cancer (Rawat et al., 2021). However, DOX administration frequently leads to left ventricular dysfunction and cardiac failure in patients and mice (Kong et al., 2022; Yuan et al., 2023), severely restricting its application. Therefore, it is essential to investigate innovative strategies for alleviating DOX-induced cardiotoxicity.

A recent study elucidated the molecular mechanism underlying DOX-induced cardiotoxicity (Rawat et al., 2021; Kong et al., 2022), with researchers agreeing that it primarily results from autophagy activation (Yuan et al., 2023), oxidative stress (Kong et al., 2022), and DNA and mitochondrial damage (Mizuta et al., 2020; Wu et al., 2023). Nevertheless, a more profound understanding of the role of autophagy in DOX-induced cardiac toxicity is required.

Autophagy is a highly conserved catabolic process responsible for degrading most long-lived or aggregated proteins that promote intracellular material recycling (Deretic and Kroemer, 2022; Yamamoto et al., 2023). Recent studies have demonstrated the impact of autophagy on DOX-induced cardiac toxicity (Li et al., 2016). A prevailing consensus exists that DOX-induced abnormal lysosomal acidification leads to the accumulation of autolysosomes (Li et al., 2016). However, the effect of autophagy on DOX-induced cardiac toxicity remains controversial. Inhibition of autophagosome formation by Beclin-1 deficiency in the heart alleviated DOX-induced cardiac dysfunction, cardiomyocyte apoptosis, and oxidative stress, whereas Beclin-1 overexpression aggravated these detrimental effects (Li et al., 2016). In the heart of DOX-treated zebrafish, atg7 overexpression reversed cardiac function decline in the late phase of DOX-induced cardiac dysfunction (Wang Y. et al., 2021). Autophagy protein 5 (ATG5) is essential in autophagosome elongation (Cicchini et al., 2015). ATG5 protein conjugates with Atg12 and Atg16L1 to form a complex that promotes the extension of the phagophore membrane in autophagic vesicles; therefore, ATG5 is indispensable for autophagosome formation (Iriondo et al., 2023). Recent reports have indicated that cardiomyocyte-specific Atg5-deficient mice develop left ventricular hypertrophy that progresses to heart failure, which ultimately leads to premature death (Taneike et al., 2010). Moreover, ATG5 knockdown diminishes mitochondrial abundance and disrupts calcium ion cycling, thereby reducing the cardiac capacity in mice and humans (Ljubojevic-Holzer et al., 2022). ATG5 deficiency decreases the fibrotic effect of TGF-β in primary human atrial myofibroblasts (Ghavami et al., 2015). A recent study investigated the role of ATG5 in DOX-induced cardiotoxicity in rats. It was observed that ATG5 knockdown by rAAV-dnATG5 to inhibit autophagosome formation prevented DOX cardiotoxicity by maintaining normal autophagic flux and decreasing lysosomal demand (Montalvo et al., 2020). rAAV-dnATG5 alleviated acute DOX-induced mitochondrial dysfunction and ROS generation, thereby improving cardiac function in DOX-treated rats; however, prolonged autophagy inhibition did not influence DOX-induced rAAV-dnATG5 (Montalvo et al., 2020). Moreover, the effects and mechanism of ATG5 in DOX-induced cardiac dysfunction in mice remained unclear.

GATA4 is a transcription factor that regulates cardiac hypertrophy and survival (Liang and Molkentin, 2002; Oka et al., 2006). Reduced GATA4 expression leads to myocardial apoptosis and cardiac dysfunction (Oka et al., 2006). GATA4 transcript and protein levels are reduced in DOX-treated hearts, and GATA4 overexpression counteracts DOX-induced cardiotoxicity (Aries et al., 2004). The anti-apoptotic protein bcl-2 is an essential target of GATA4; GATA4 knockdown reduces *bcl-2* gene expression, whereas GATA4 overexpression upregulates bcl-2 in neonatal rat ventricular cardiomyocytes and the heart (Kobayashi et al., 2006). Recent studies have revealed that GATA4 protein expression is regulated by autophagy (Song et al., 2021; Chen et al., 2022); however, whether ATG5-mediated autophagy is responsible for GATA4 degradation and promotes DOX-induced cardiotoxicity remains unclear.

This study discovered that blocking autophagy by rAAV9-shATG5 improves DOX-induced myocardial fibrosis and oxidative stress by restoring GATA4, suggesting that ATG5 is a potential target to reverse the decline in cardiac function caused by DOX.

## Materials and methods

### Antibodies and reagents

Anti-Tubulin (66031-1-Ig), anti-TGF-β (21898-1-AP), anti-Bax (50599-2-Ig), and GATA4 (19530-1-AP) were acquired from Proteintech (Wuhan, Hubei, China). Anti-ATG5 (12994T), anti-P62 (18420-1-AP), and anti-cleaved caspase 3 (9664T) were obtained from Cell Signaling Technology (Danvers, MA, United States). Anti-LC3B (L8918) was obtained from Sigma-Aldrich, and anti-phosphorylated smad2/3 (K009346P), anti-smad2 (K005398P), and anti-smad3 (K000497P) were procured from SolarBio (Beijing, China). DOX, wheat germ agglutinin (WGA), and dehydroergosterol (DHE) were provided by Sigma-Aldrich (St Louis, MO, United States). Bafilomycin A1 (BafA1) was obtained from Meilunbio (Dalian, Liaoning, China), and the TUNEL assay kit was acquired from Intergen (NY, United States). Hematoxylin and eosin and Masson’s trichrome assay kits were obtained from Solarbio (Beijing, China) and TRIzol from Invitrogen (Carlsbad, CA, United States).

### Animal and treatment

Male C57BL/6 mice (8–10 weeks old) were acquired from Liaoning Changsheng Biotechnology (Benxi, Liaoning, China) and housed in plastic cages in a pathogen-free environment with a 12-h light/dark cycle/day. The rAAV vector (serotype 9, AAV-9) containing the *shRNA-ATG5, ATG5,* and *GATA4* genes driven by the cardiac troponin T promoter was constructed and then delivered into mice by a single intravenous injection for 3 weeks (5 × 10^11^ μg/mg, Shandong Weizhen Biotechnology). Then, the mice were intravenously administered DOX (Cat# D1515, Sigma-Aldrich, United States) at 5 mg/kg/week or vehicle for 4 weeks. To assess autophagic flux by Western blotting, BafA1 (1.5 mg/kg) was administered to mice 2 h before sacrifice. All animal experiments were approved by the Animal Experimentation Ethics Committee of Binzhou Medical University and agreed with the US National Institutes of Health (National Institutes of Health Publication No. 85–23, revised 1996).

### Echocardiography

The mice were placed in an anesthesia induction box, anesthetized with 1.5% isoflurane, and maintained at a heart rate of 450–500 beats/min. Transthoracic echocardiography was performed on the mice using a Vevo 1100 ultrasound machine and a 30 MHz transducer (VisualSonics, Canada). A two-dimensional guided M-mode trace from the parasternal short axis crossing the papillary muscle was recorded. Left ventricular ejection fraction (EF%) and fractional shortening (FS%) at systole and diastole were analyzed.

### Histopathology

Cardiac tissue was fixed overnight in 4% paraformaldehyde (Servicebio, Wuhan, China), embedded in paraffin, and cut into 5 µm sections. Masson’s trichrome staining was performed following the instructions provided by the reagent supplier (Solarbio, Beijing, China) to evaluate the situation of cardiac fibrosis.

### DHE (dehydroergosterol) staining

Fresh cardiac tissue was quickly frozen at −20 °C and immediately cut into 10 µm sections. The sections were stained with 10 µM DHE (catalog number D7008, Sigma-Aldrich, Santa Clara, CA, United States) in a dark wet chamber at room temperature for 30 min at 37°C and then washed with PBS. Images were obtained in 10–20 random fields using a fluorescence microscope (BX51, OLYMPUS, Japan), and the fluorescence intensity was measured using Image-Pro Plus software (version 3.0).

### WGA staining

The cardiac tissue was fixed overnight in 4% paraformaldehyde, embedded in paraffin, and cut into 5 µm sections. It was heated in citric acid after dewaxing in xylene and different ethanol concentrations to induce antigen retrieval. When the section was cooled, the WGA solution was added to cover the cardiac tissue surface for 30 min in the dark at 37°C. The sections were washed thrice with PBS, and the images were captured and photographed using a fluorescence microscope (BX51, OLYMPUS, Japan).

### Immunofluorescent staining

Cardiac tissue was fixed in 4% paraformaldehyde, dehydrated in 30% sucrose overnight, embedded in Tissue-Tek OCT (SAKURA, Japan), and quickly frozen at −20°C. The samples were cut into 10 µm sections, and the sections were permeabilized in PBS buffer containing 1% BSA and 1‰ TritonX-100 for 15 min, then blocked with goat serum (1:200, Meilunbio, Dalian, China) for 1 h at room temperature. The sections were incubated overnight with anti-actinin (1:500, Sigma-Aldrich) and anti-LC3B (1:100, Proteintech) at 4 °C and with the appropriate secondary antibody for 1 h. Images were obtained using a laser scanning confocal microscope (TCS SP8; Leica Microsystems).

### Tunel staining

Apoptosis in the cardiac sections was detected using the *in situ* cell death detection kit (TUNNEL fluorescence FITC kit, Roche) following the manufacturer’s instructions.

Frozen heart sections were permeabilized in 0.1% Triton x-100 and resolved in sodium citrate buffer. Apoptotic cells were stained using an *in situ* cell death detection kit (Roche), and nuclei were stained blue with DAPI. Total cells and tunnel-positive cells were automatically counted using Image-Pro Plus software. The apoptosis rate was defined as the ratio of apoptotic cells to the total cells.

### Cell culture and treatment

H9c2 cells were acquired from ProCell Life Science and Technology (Wuhan, China). H9c2 cells were grown in DMEM F12 medium containing 10% fetal bovine serum (Gibco, United States), 2 mM L-glutamine, 0.1 mM norepinephrine, and 100 μg/mL penicillin/streptomycin at 37°C in a humidified atmosphere of 5% CO_2_. H9c2 cells were incubated with DOX (1.0, 2.5, and 5.0 µM) for 12 h and subjected to Western blotting for drug treatment. For lysosome inhibitor Baf A1 treatment, H9c2 cells were incubated with Baf A1 (50 nM) for 2 h before the cells were lysed.

### Autophagic flux detection

H9c2 cells were transfected with RFP-GFP-LC3 lentivirus (0.1 × 10^8^/mL MOI = 10, Hanheng Bio, Shanghai) for 24 h and then treated with DOX (1 µM) for 12 h. After the cells were stained with DAPI for 5 min, autophagic flux was detected using a laser scanning confocal microscope (TCS SP8; Leica Microsystems). The red dots represented autophagolysosomes, and the green dots represented autophagosomes.

### Immunofluorescence

H9c2 cells were fixed with 4% paraformaldehyde for 15 min, permeabilized with 1‰ TritonX-100 for 15 min, and blocked with 3% bovine serum albumin (Solarbio) for 1 h at room temperature. After washing thrice with PBS, the cells were incubated with LC3 and GATA4 antibodies, both at a dilution of 1:200 overnight at 4°C. After rigorous washing, the cells were incubated with secondary antibodies conjugated with Alexa Fluor 594 nm (1:200; Zenbio) or Alexa Fluor 488 nm (1:200; Zenbio) for 1 h at room temperature. After the cells were washed with PBS thrice and mounted using Antifade Mounting Medium with DAPI (Beyotime), all images were obtained using a laser scanning confocal microscope (TCS SP8; Leica Microsystems).

### Co-immunoprecipitation

Cells were lysed with IP lysis buffer [50 mM Tris-HCl (pH 8.0), 150 mM NaCl, 1% NP-40, 1% SDS, and 0.5% sodium deoxycholate] supplemented with protease inhibitor cocktail (Roche) on ice for 20 min. The lysates were precleared after the samples were centrifuged at 12,000 × g for 15 min. They incubated with the indicated primary antibodies overnight at 4°C and then with protein A/G magnetic beads (Thermo Fisher Scientific) with gentle shaking at 4°C overnight. The beads were washed five times with washing buffer, and the samples were harvested for Western blotting.

### Western blots

Cardiac tissue or cells were lysed with ice-cold RIPA buffer containing a 1% protease inhibitor cocktail for 30 min. The samples were centrifuged at 4 °C for 15 min. The protein concentration was determined using a BCA assay kit (Thermo Scientific). Proteins (20–50 mg) were subjected to SDS-PAGE gel to separate the sample proteins and then transferred to the PVDF membrane (Merck, United States). After blocking with 5% skim milk (Solarbio, D8340) for 1 h at room temperature, the membrane was incubated with primary antibodies at 4 °C overnight. After washing with TBST thrice, the membrane was incubated with a horseradish peroxidase-conjugated secondary antibody (1:2,500, Proteintech) at room temperature. Enhanced chemiluminescence with the Pierce^®^ Western blotting Substrate (Thermo Fisher Scientific) and Gel-Pro 4.5 Analyzer determined protein expression.

### Quantitative polymerase chain reaction (qRT-PCR)

TRIzol (Invitrogen, Carlsbad, CA) was used to extract RNA, and Hifair^®^ III 1st Strand cDNA Synthesis Kit (Yeasen, China) was used to reverse RNA into double-stranded cDNA followed by qRT-PCR amplification using Hieff UNICON^®^ Universal Blue qPCR SYBR Green Master Mix (Yeasen, China) and the 7,500 Fast Real-Time PCR Systems (Applied Biosystems, United States). GAPDH was used as an internal control for mRNA, and the 2^−ΔΔCT^ method was used to analyze relative gene expression levels. The primers were used as follows: *GAPDH* (Forward: GGT​TGT​CTC​CTG​CGA​CTT​CA; Reverse: GGT GGT​CCA​GGG​TTT​CTT​ACT​C); *ANP* (Forward: TAC​AGT​GCG​GTG​TCC​AAC​ACA​G; Reverse: TGC​TTC​CTC​AGT​CTG​CTC​ACT​C); *Collagen I* (Forward: GAGTACTGGAT CGACCCTAACCA; Reverse: GAC​GGC​TGA​GTA​GGG​AAC​ACA); *Collagen III* (Forward: TCC​CCT​GGA​ATC​TGT​GAA​TC; Reverse: TGA​GCG​AAT​TGG​GGA​GAA​T); *ATG5* (Forward: AGA​GTC​AGC​TAT​TTG​ACG​TTG​G; Reverse: TGGACAGTGTAGAAG GTCCTTTT); *Beclin* (Forward: GAG​CCA​TTT​ATT​GAA​ACT​CGC​CA; Reverse: CCTCC CCGATCAGAGTGAA); *LC3B* (Forward: CGC​TTG​CAG​CTC​AAT​GCT​AAC; Reverse: TCT​CTC​ACT​CTC​GTA​CAC​TTC​G).

### Statistical analyses

Data are expressed as the mean ± standard error of the mean (SEM). Multiple comparisons were performed using one-way analysis of variance (ANOVA; Tukey’s *post hoc* test), and two group comparisons were performed using a *t*-test. A *P* < 0.05 was considered statistically significant. All data were analyzed using GraphPad Prism software (version 6.07; GraphPad Software Inc.).

## Results

### The effects of DOX on autophagic flux on the heart of mice

**FIGURE 1:**
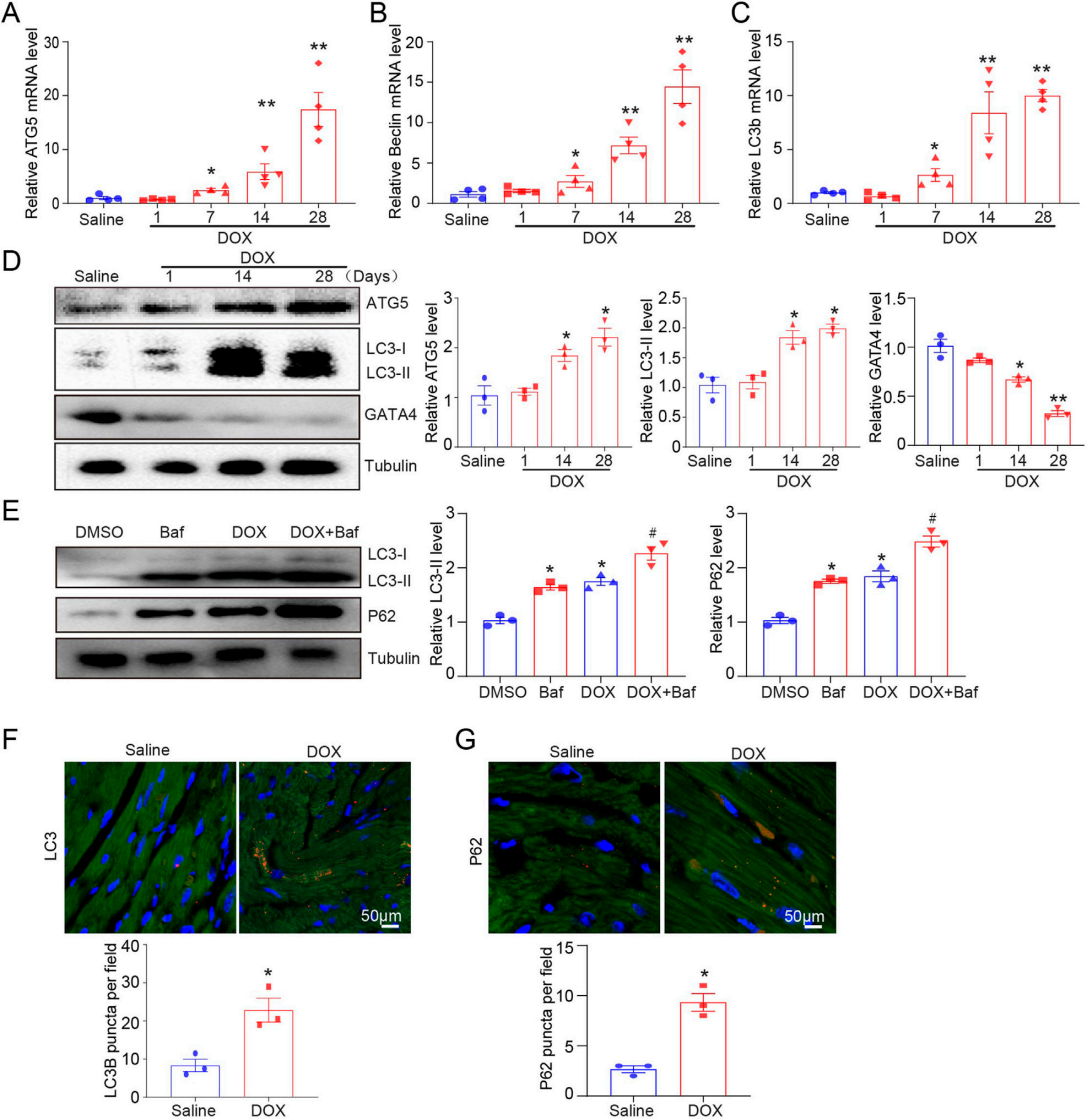
Dynamic autophagy signaling in DOX-induced cardiotoxicity model of mice. **(A–C)** Quantifying *ATG5, beclin, and LC3B* gene expression by quantitative real-time polymerase chain reaction. N = 4/group. **(D)** Representative Western blotting and quantification indicating changes in ATG5, GATA4, and LC3-II protein expression in the hearts of DOX-treated mice (n = 3/group). **(E)** Changes in P62 and LC3-II protein expression in the hearts of DOX-treated mice in the presence or absence of baflomycin A1. BafA1 (1.5 mg/kg) was administered to the mice 2 h before sacrifice (n = 3/group). **(F, G)** Immunohistochemical staining of LC3B and P62 in the hearts of mice treated with vehicle or DOX. Quantification is illustrated below (n = 3/group). Data are presented as the mean ± SEM. Statistical significance was determined by one-way ANOVA with Tukey’s *post hoc* test, and Student’s t-test was used for **(F, G)**. *P < 0.05, **P < 0.01 versus the saline group; ^#^ *P* < 0.05, versus the DOX group.

Mice received tail intravenous injections of DOX for 1, 7, 14, and 28 days, and the mRNA levels of *ATG5*, *Beclin-1*, and *LC3B* in the heart were determined by qPCR. DOX stimulated autophagy initiation (by upregulating *ATG5*, *Beclin-1*, and *LC3b* genes), simultaneously increasing the protein expression of ATG5 and LC3-II and reducing GATA4 protein expression (Figures 1A–D). To identify whether the increase in LC3B expression caused by DOX indicated the promotion of autophagy, a lysosome inhibitor, bafilomycin, was used to inhibit the fusion of autophagosomes and lysosomes, and the effect of DOX on LC3-II expression was determined. DOX increased LC3-II and P62 expression in the presence or absence of bafilomycin (Figure 1E). The increase in P62 protein level after DOX treatment indicated that the degradation of P62 by autophagy was disrupted, suggesting that DOX increased autophagosome formation but reduced autophagic degradation in the heart. Consistent with the results for LC3 and P62 protein levels, we observed a significant increase in the abundance of LC3 and P62 puncta in DOX-treated hearts (Figures 1F, G).

### DOX inhibited the fusion of autophagosomes and lysosomes in H9c2 cells

**FIGURE 2:**
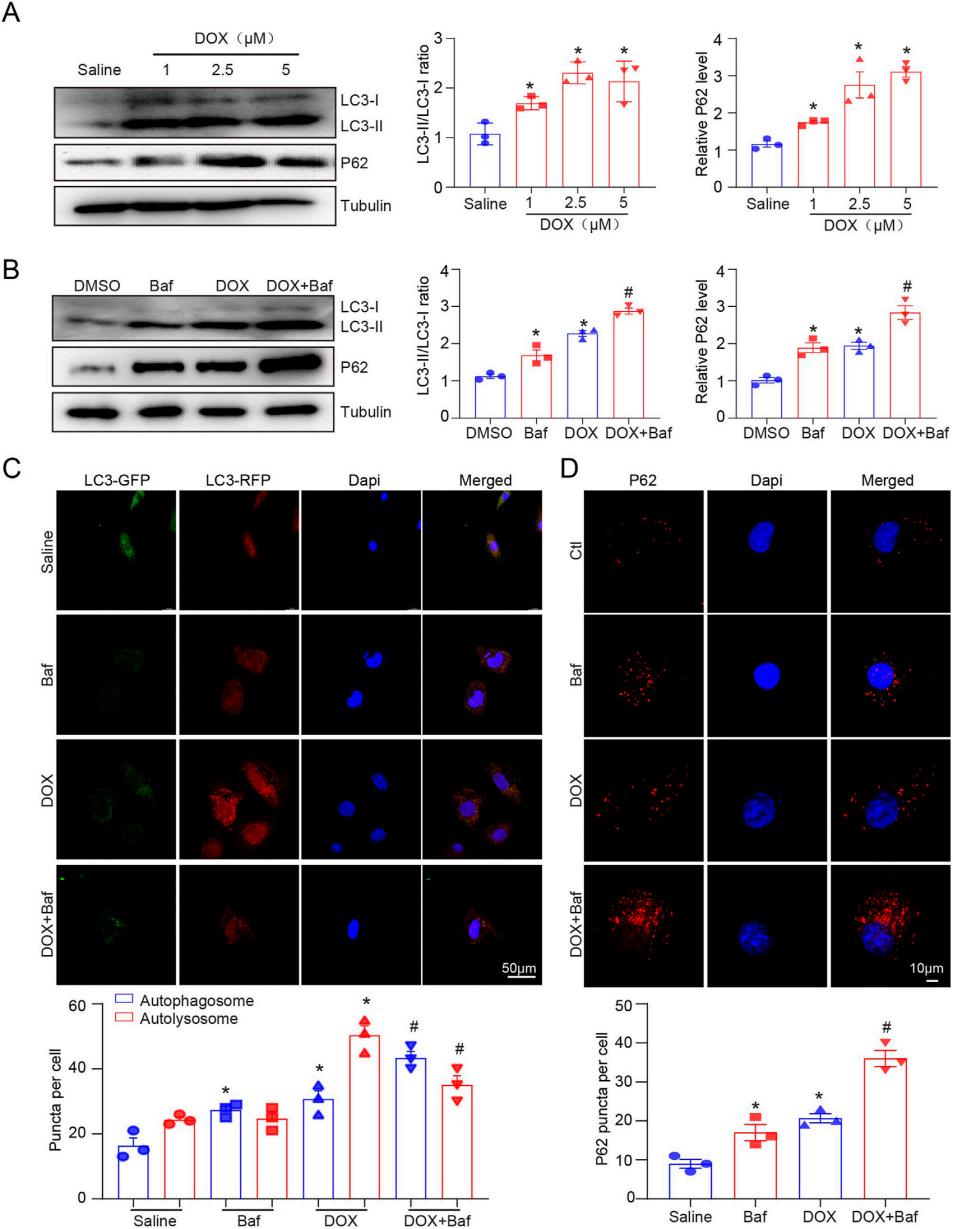
The effects of DOX on autophagic flux in H9c2 cells. **(A)** Representative Western blotting and quantification illustrating the alteration in LC3-II and P62 protein expression in H9c2 cells after DOX (1.0, 2.5, and 5.0 µM) treatment for 12 h, n = 3/group. **(B)** LC3-II and P62 protein expression in 2.5 µM DOX-treated H9c2 cells in the presence or absence of 50 nM BafA1 (n = 3/group). **(C)** H9c2 cells were transiently transfected with mRFP-green fluorescence protein (GFP)-LC3 adenovirus and treated with DOX for 12 h. Representative images of the GFP-LC3 and mRFP-LC3 puncta are presented. Quantification of the yellow puncta (autophagosomes) and red puncta (autolysosomes) is below (n = 3/group). **(D)** Immunofluorescence staining of P62 in H9c2 cells treated with DOX in the presence or absence of BafA1 and the quantification is presented below (n = 3/group). Data are presented as the mean ± SEM. Statistical significance was determined by one-way ANOVA with Tukey’s *post hoc* test, *P < 0.05, versus the saline group; ^#^ *P* < 0.05, versus the DOX group.

We measured LC3-II and P62 protein expression and autophagic flux in H9c2 cells after treatment with 1, 2.5, and 5 µM DOX for 12 h. Similarly, LC3B protein expression increased dose-dependent after DOX treatment (Figure 2A). Baf A1 was used to block the fusion of autophagosomes and lysosomes to further confirm the effect of DOX on autophagy. We identified that LC3-II and P62 expression significantly increased after Baf A1 or DOX treatment (Figure 2B), owing to the accumulation of autolysosomes and inhibition of autophagic flux. DOX significantly increased autophagosome and autolysosome generation in RFP-GFP-LC3 expressed H9c2 cells (Figure 2C). Consistent with our *in vivo* results, p62 puncta in the left ventricular cardiac tissues were increased in DOX-treated H9c2 cells (Figure 2D). To identity whether the fusion of autophagosomes and lysosomes is blocked, we performed immunofluorescent staining to detect the co-localization of LC3-II (an autophagosomal marker) and LAMP1(a lysosomal marker), and found that DOX inhibited LAMP1 and LC3 co-localization, which was further aggravated in the presence of bafilomycin (Supplementary Figure S1A). These results indicated that DOX increased autophagosome formation but inhibited the fusion of autophagosomes and lysosomes in H9c2 cells.

### rAAV9 delivers shRNA-ATG5 alleviated DOX-induced cardiac dysfunction and fibrosis

**FIGURE 3:**
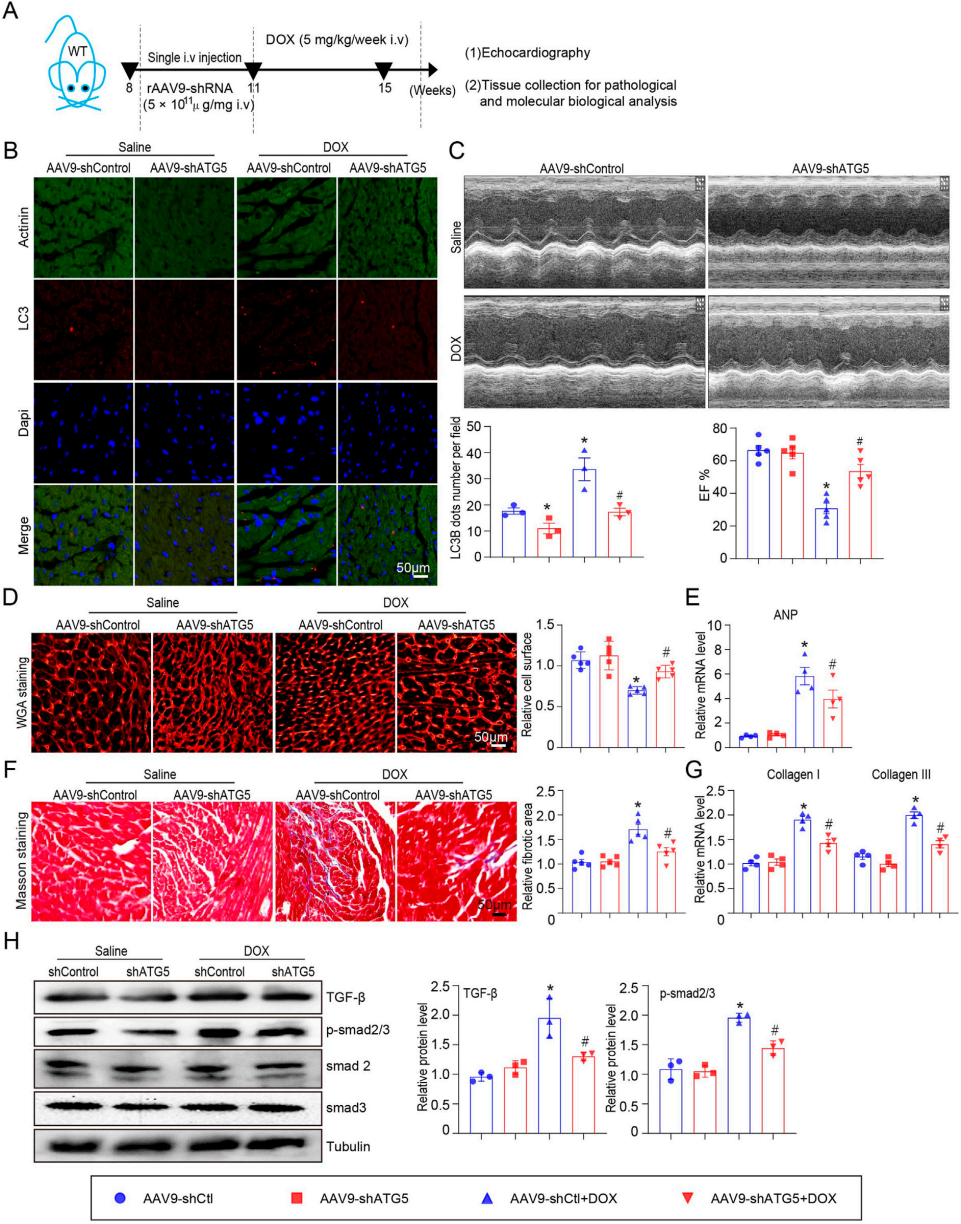
ATG5 knockdown inhibited DOX-induced cardiac dysfunction and fibrosis. **(A)** Mice were intravenously administered a single rAAV9-shRNA-ATG5 for 3 weeks and then with DOX for another 4 weeks. **(B)** Immunohistochemical staining of LC3B and quantification are presented (n = 3/group). **(C)** The dynamics of ejection fraction (EF)% in mice was examined using a high-frequency echo system (n = 5). **(D)** Representative images of WGA staining illustrating myocardial cell size in DOX-treated hearts in the presence or absence of rAAV9-shRNA-ATG5 infection (n = 5/group). **(E)** Quantification of *ANP* gene expression using quantitative real-time polymerase chain reaction. N = 4/group. **(F)** Representative Masson’s staining and quantitative analysis of cardiac fibrosis in heart tissues (n = 5). **(G)** Evaluation of collagen I and III gene transcript expression using quantitative real-time polymerase chain reaction (n = 4). **(H)** Representative Western blotting images and quantitative analysis of TGF-β, p-smad2/3, smad2, and smad3 in the heart tissues (n = 3). Data are presented as the mean ± SEM. Statistical significance was determined by one-way ANOVA with Tukey’s *post hoc* test, *P < 0.05, versus the AAV9-shCtl group; ^#^ *P* < 0.05, versus the AAV9-shCtl + DOX group.

The role of ATG5-mediated autophagy in DOX-induced cardiac dysfunction was evaluated. Mice received single intravenous injection with rAAV9-shRNA-ATG5 for 3 weeks to knock down ATG5 and were then subjected to DOX for another 4 weeks (Figure 3A). Immunohistochemical staining indicated that rAAV9-shRNA-ATG5 injection successfully reduced LC3B expression in the heart compared with rAAV9-shRNA-Control (Figure 3B). Furthermore, we observed that the DOX-induced increase in LC3B was inhibited by rAAV9-shRNA-ATG5 (Figure 3B). Heart function was assessed using echocardiography. We discovered that DOX treatment for 4 weeks significantly induced cardiac dysfunction, reflected by reduced left ventricular EF%, and knockdown ATG5 by rAAV9 markedly alleviated DOX-induced cardiac dysfunction (Figure 3C). It has been suggested that DOX administration results in cardiac hypotrophy and fibrosis (Liao et al., 2021). DOX reduced cardiomyocyte size, and this effect was partly attenuated by rAAV9-siRNA-ATG5 (Figure 3D). Although the number of cardiomyocytes in DOX group seems to be more than Saline group, this was due to reduced cell size caused by DOX but not the proliferative effect. CCK-8 assay results showed that genetic knockdown or overexpressing ATG5 had no effect on DOX reduced cell viability in H9c2 cells (Supplementary Figures S1B, C). Furthermore, *ANP* mRNA expression, a heart failure biomarker, increased in DOX-treated hearts and was inhibited when ATG5 was knocked down by rAAV9-shRNA-ATG5 (Figure 3E). Masson’s trichrome staining and qPCR results revealed DOX-induced cardiac fibrosis and increased collagen I and III mRNA expression. This effect was partially alleviated by rAAV9-siRNA-ATG5 (Figures 3F,G). The signaling pathway that mediates cardiac fibrosis was examined by Western blotting. DOX enhanced TGF-β and p-smad2/3 protein levels, which were dramatically inhibited by rAAV9-siRNA-ATG5 (Figure 3H).

### Knockdown ATG5 inhibited DOX-induced oxidative stress and myocardial apoptosis

**FIGURE 4:**
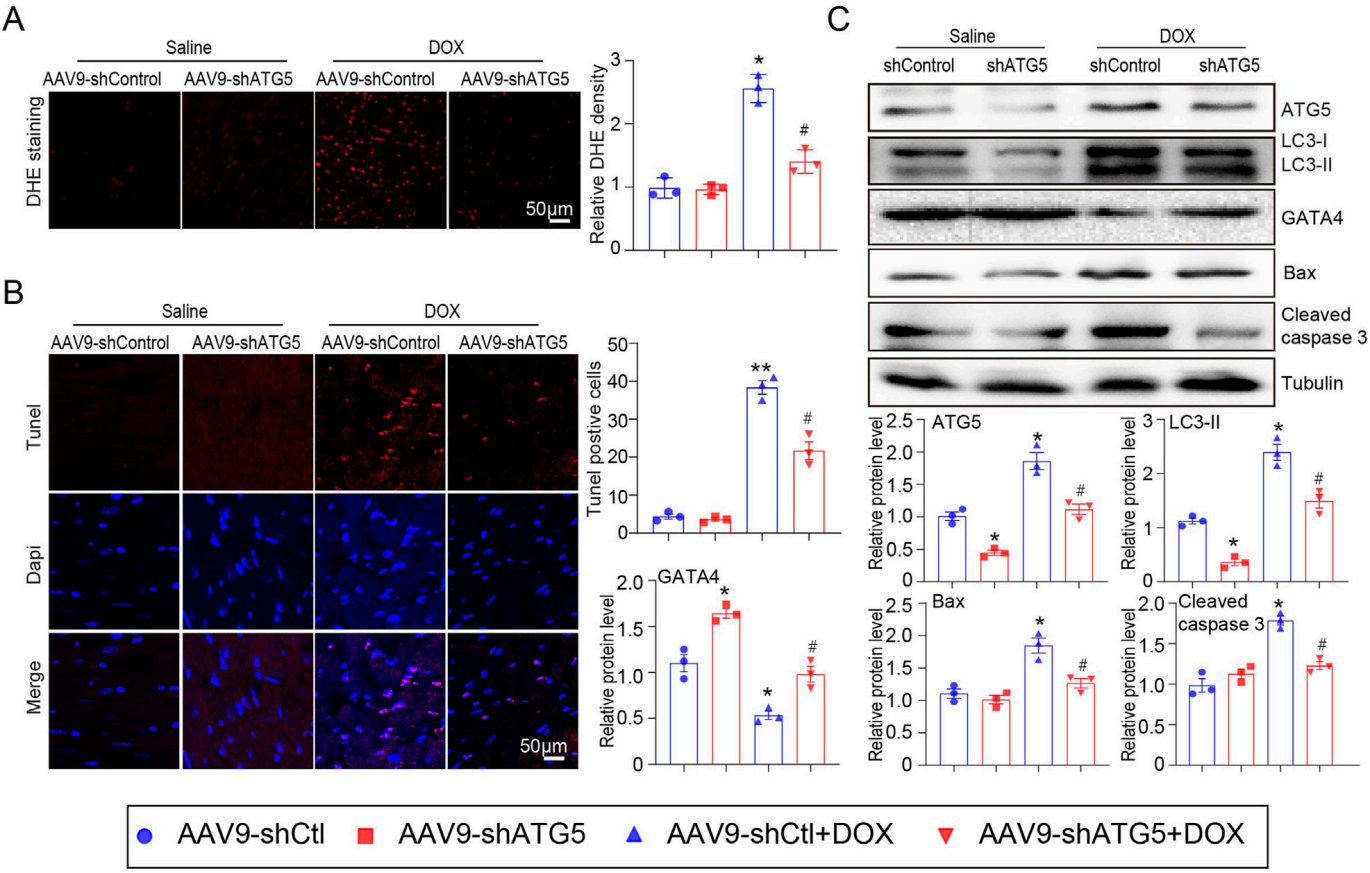
ATG5 knockdown alleviated DOX-induced oxidative stress and apoptosis. **(A)** Representative DHE staining images and quantitative results demonstrating ROS generation in the heart tissues (n = 3). **(B)** Representative images of Tunel staining and the quantitative results indicating the myocardial apoptosis in heart tissues (n = 3). **(C)** Representative Western blotting images and quantitative analysis of *ATG5, LC3-II, GATA4, Bax,* and *cleaved caspase-3* in the heart tissues (n = 3). Data are presented as the mean ± SEM. Statistical significance was determined by one-way ANOVA with Tukey’s *post hoc* test, *P < 0.05, versus the AAV9-shCtl group; ^#^ *P* < 0.05, versus the AAV9-shCtl + DOX group.

ROS generation in the hearts of mice was detected by DHE staining. The results indicated that DOX treatment significantly increased ROS generation in the heart, as reflected by the enhanced red fluorescence intensity of the heart compared with that of the saline-treated heart (Figure 4A). Excess ROS accumulation can induce myocardial apoptosis, so the heart sections were subjected to Tunnel staining to detect cell apoptosis. DOX significantly induced myocardial apoptosis, which was partially inhibited by rAAV9-shRNA-ATG5 (Figure 4B). Since DOX treatment can reduce GATA4 expression, which can upregulate the anti-apoptotic genes *Bcl-2 and Bcl-xL* in the heart (Aries et al., 2004) and be degraded by autophagy (Chen et al., 2022), its protein expression was determined by Western blotting. We observed that rAAV9-shRNA-ATG5 successfully knocked down ATG5 and reduced LC3-II protein expression, leading to autophagy inhibition (Figure 4C). Meanwhile, GATA4 protein expression was reduced by DOX, but the pro-apoptotic protein Bax and cleaved caspase 3 protein expression were increased by DOX treatment. These effects were significantly inhibited by rAAV9-shRNA-ATG5, suggesting that ATG5 knockdown controls GATA4 protein levels by inhibiting autophagic degradation (Figure 4C).

### Overexpressing ATG5 aggravated DOX-induced cardiac dysfunction and fibrosis

**FIGURE 5:**
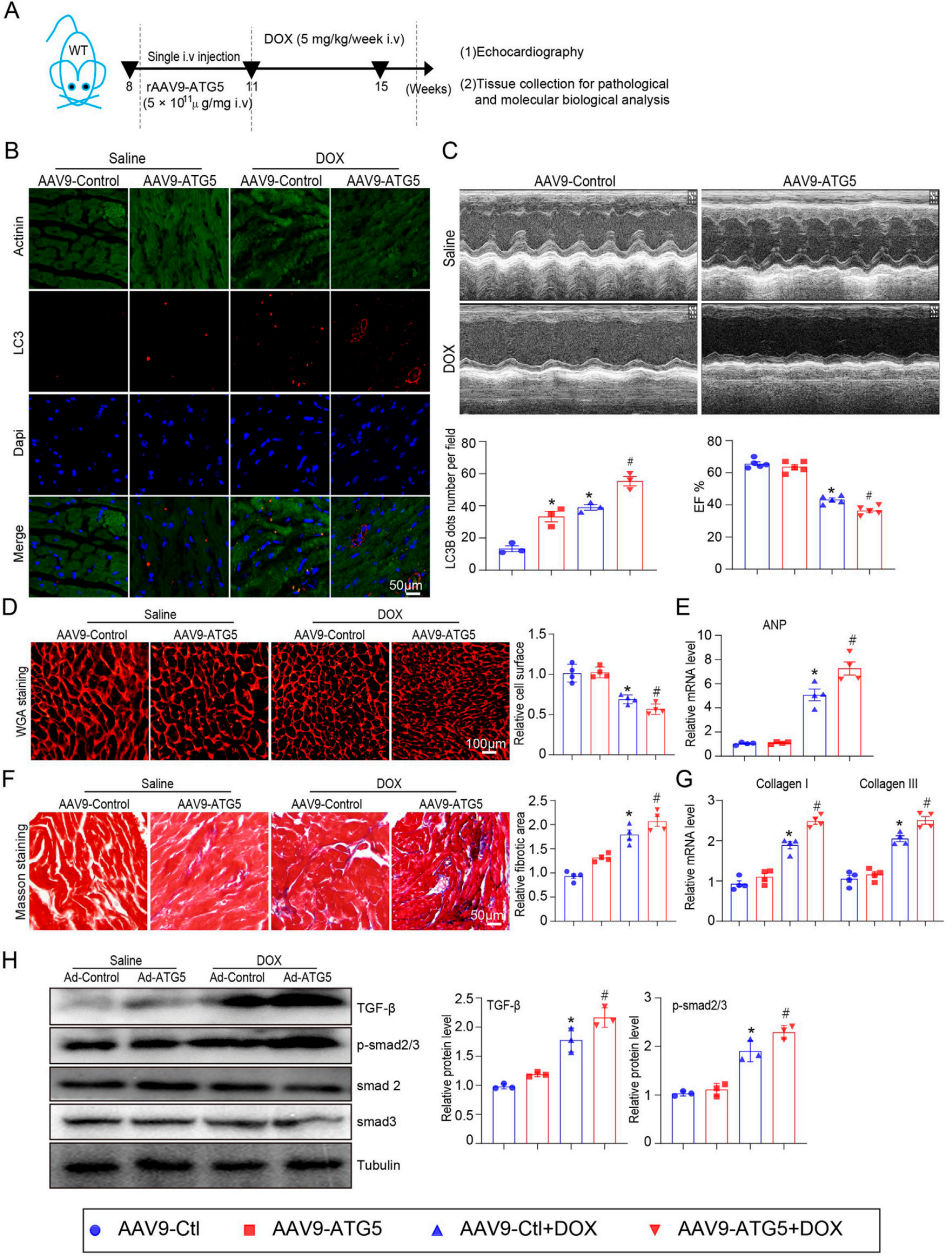
ATG5 overexpression aggravated DOX-induced cardiac dysfunction and fibrosis. **(A)** Mice were intravenously administered a single rAAV9-ATG5 for 3 weeks and then with DOX for another 4 weeks. **(B)** Immunohistochemical staining of LC3B and quantification are presented (n = 3 per group). **(C)** The dynamics of ejection fraction (EF)% in mice was examined using a high-frequency echo system (n = 5). **(D)** Representative images of WGA staining indicating the myocardial cell size in DOX-treated hearts in the presence or absence of rAAV9-ATG5 infection (n = 4/group). **(E)** Quantification of *ANP* gene expression using quantitative real-time polymerase chain reaction. N = 4/group. **(F)** Representative Masson’s staining and quantitative analysis of cardiac fibrosis in heart tissues (n = 4). **(G)** Evaluation of *collagen I and III* gene transcript expression using quantitative real-time polymerase chain reaction (n = 4). **(H)** Representative images of Western blotting and quantitative analysis of TGF-β, p-smad2/3, smad2, and smad3 in heart tissues (n = 3). Data are presented as the mean ± SEM. Statistical significance was determined by one-way ANOVA with Tukey’s *post hoc* test, *P < 0.05, versus the AAV9-Ctl group; ^#^ *P* < 0.05, versus the AAV9-Ctl + DOX group.

Mice were infected with rAAV9 expressing ATG5 for 3 weeks to increase ATG5 expression in the heart and then subjected to DOX for another 4 weeks (Figure 5A). Injection of rAAV9 expressing ATG5 induced autophagy and increased LC3B-positive cells in both saline- and DOX-treated hearts (Figure 5B). Echocardiography results indicated that ATG5 overexpression in the heart did not affect left ventricular EF% at the basal condition but aggravated DOX-induced cardiac dysfunction (Figure 5C). In contrast to the results obtained in ATG5 knockdown mice, enforced ATG5 expression further aggravated DOX-induced cardiac hypotrophy, as reflected by the reduced cardiomyocyte size (Figure 5D). *ANP* mRNA expression in the DOX + rAAV9- ATG5 group was higher than in the DOX + rAAV9-Control group, indicating more severe cardiac injury in the ATG5 overexpressing heart (Figure 5E). We performed Masson’s trichrome staining and qPCR experiments to address whether ATG5 overexpression in the heart could further increase DOX-induced cardiac fibrosis. The results revealed DOX-induced cardiac fibrosis and increased collagen I and III mRNA expression, which were further aggravated by rAAV9-ATG5 (Figures 5F, G). The increased protein expression of TGF-β and p-smad2/3 induced by DOX was further increased by rAAV9-ATG5 (Figure 5H).

### ATG5 overexpression aggravated DOX-induced oxidative stress and myocardial apoptosis

**FIGURE 6:**
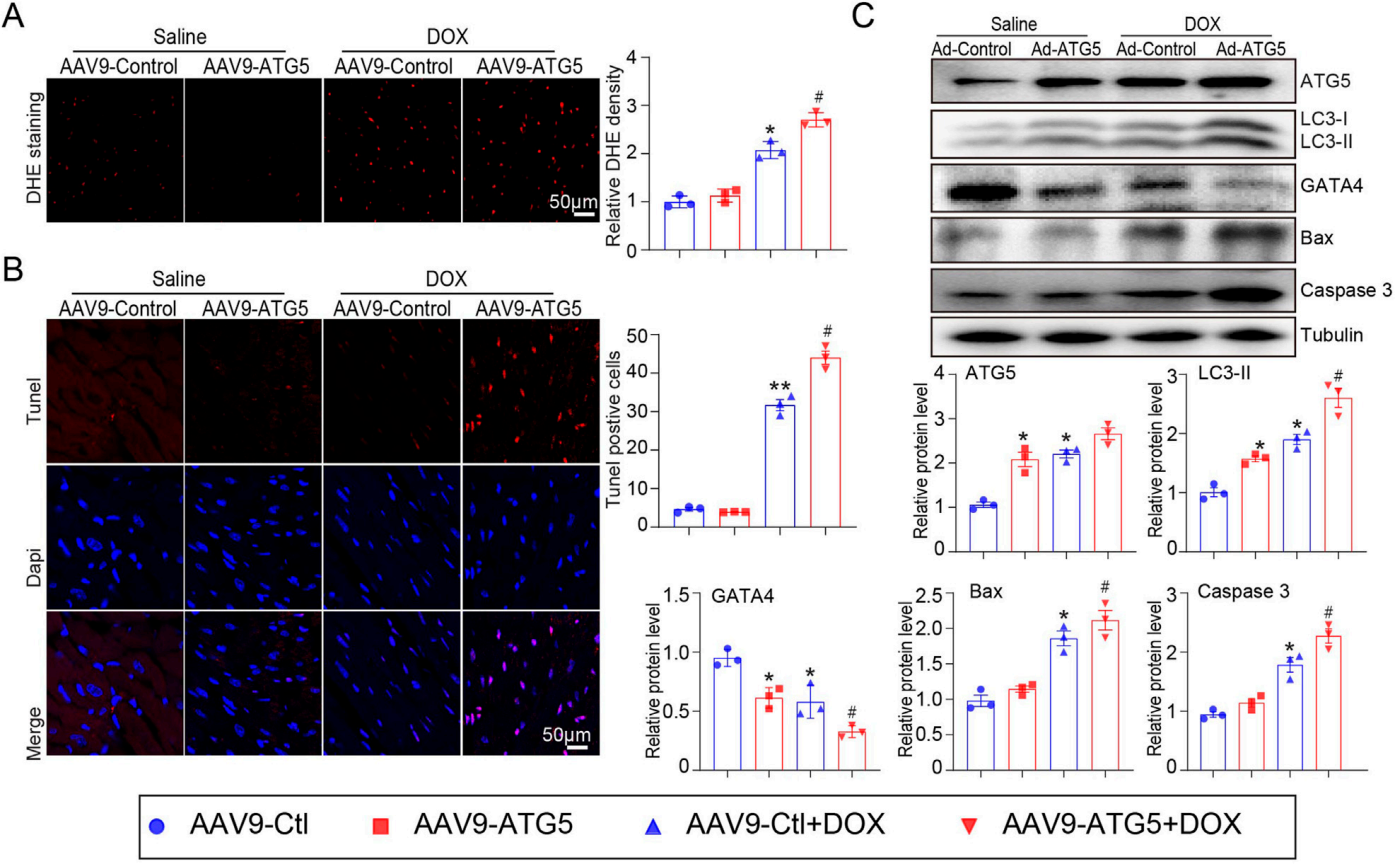
ATG5 overexpression aggravated DOX-induced cardiac oxidative stress and apoptosis. **(A)** Representative images of DHE staining and quantitative results indicating ROS generation in the heart tissues (n = 3). **(B)** Representative images of Tunel staining and the quantitative results displaying the myocardial apoptosis in heart tissues (n = 3). **(C)** Representative Western blotting images and quantitative analysis of ATG5, GATA4, LC3-II, Bax, and cleaved caspase-3 in the heart tissues (n = 3). Data are presented as the mean ± SEM. Statistical significance was determined by one-way ANOVA with Tukey’s *post hoc* test, *P < 0.05, versus the AAV9-Ctl group; ^#^ *P* < 0.05, versus the AAV9-Ctl + DOX group.

To investigate the effect of ATG5-mediated autophagy on oxidative stress and apoptosis, DHE and Tunnel staining were performed. Our results indicated that DOX significantly promoted ROS generation and induced myocardial apoptosis, which was aggravated by rAAV9-ATG5 infection (Figures 6A, B). Protein-mediated autophagy (ATG5 and LC3-II) and apoptosis (Bax and cleaved caspase 3) were determined by Western blotting. The results revealed that the expression of these proteins increased in DOX-treated hearts compared with saline-treated hearts, and overexpression of ATG5 in the heart by rAAV9-ATG5 further enhanced their expression (Figure 6C). Meanwhile, DOX reduced GATA4 protein in the heart, and the enforced expression of ATG5 by rAAV9-ATG5 further reduced its protein level, suggesting that ATG5-mediated autophagy promoted GATA4 degradation (Figure 6C).

### ATG5-mediated autophagy promoted GATA4 degradation

**FIGURE 7:**
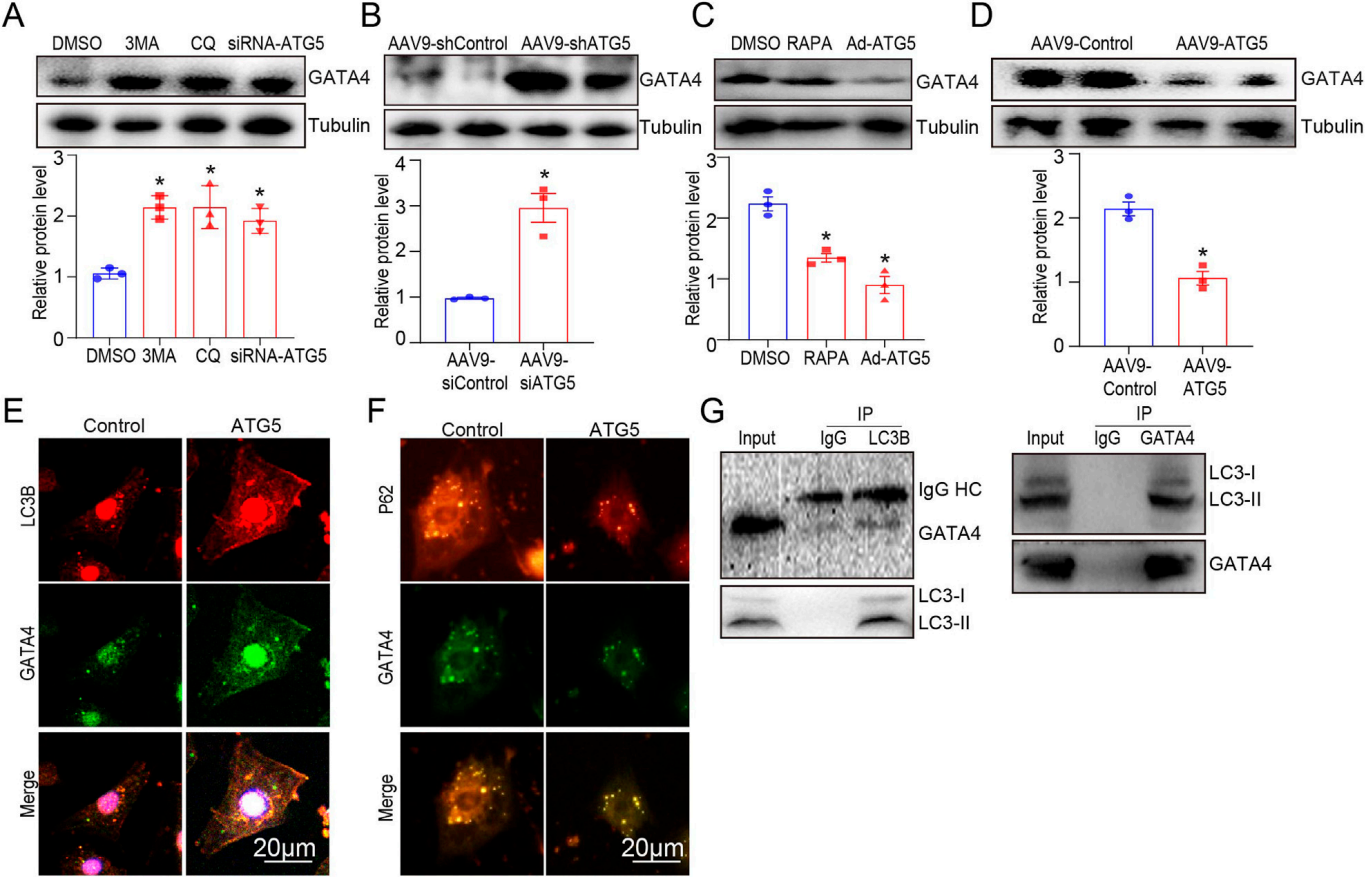
ATG5-mediated autophagy promoted GATA degradation. **(A)** GATA4 was determined by Western blotting in H9c2 cells treated with DMSO, CQ (20 μmol/L), 3-MA (5 mmol/L), and siRNA-ATG5. **(B)** Mice were intravenously administered rAAV9-shATG5 for 3 weeks, and GATA4 was determined by Western blotting. **(C)** GATA4 expression in H9c2 cells treated with DMSO, RAPA (100 nmol/L), and Ad-ATG5 was determined using Western blotting. **(D)** Mice were intravenously administered rAAV9-ATG5 for 3 weeks, and GATA4 was determined by Western blotting. **(E, F)** Immunofluorescence staining of GATA4 (green) and LC3B (red), GATA4 (green) and P62 (red) in Ad-ATG5 infected H9c2 cells. Scale bars represent 20 μm. **(G)** Co-IP analysis of the interaction between GATA4 and LC3B in H9c2 cells, following immunoprecipitation with anti-LC3B and GATA4. N = 3 for all experiments, and the data are presented as the mean ± SEM. For A and C, statistical significance was determined by one-way ANOVA with Tukey’s *post hoc* test, and For B and D, Student’s t-test was used. *P < 0.05, versus DMSO, AAV9-shCtl, or AAV9-Ctl groups.

GATA4 regulates several cardiac-specific fetal genes that protect against DOX-induced cardiotoxicity (Aries et al., 2014). Studies have revealed that autophagy activation promotes GATA4 protein degradation in cardiomyocytes (Chai et al., 2023). Therefore, we hypothesized that ATG5 controls GATA4 protein levels by altering its degradation rate by regulating autophagic flux. To test this, we examined the protein levels of GATA4 in H9c2 cells and heart tissues after autophagy inhibition and activation. We identified that blocking autophagy with chloroquine (CQ), 3-MA, and adenovirus expressing siRNA-ATG5 increased basal GATA4 protein levels in H9c2 cells (Figure 7A) and cardiac-specific ATG5 knockdown by rAAV9 significantly increased basal GATA4 protein levels in mouse hearts (Figure 7B). Conversely, we observed that enhancing autophagy flux with rapamycin (RAPA) or adenovirus expressing ATG5 decreased GATA4 protein levels in H9c2 cells (Figure 7C), which was also detected in rAAV9-ATG5 infected hearts (Figure 7D). We then performed immunofluorescence analysis and observed that the co-localization of LC3B and GATA4 was increased in ATG5 overexpressing H9c2 cells (Figure 7E). Meanwhile, the co-localization of P62 and GATA4 was reduced in ATG5 overexpressing H9c2 cells (Figure 7F). In addition, we found that LC3B co-immunoprecipitated with GATA4 (Figure 7G), suggesting that ATG5-mediated autophagy promoted GATA4 degradation.

### Restoration of GATA4 reversed the effects of ATG5 on DOX-induced myocardial dysfunction and apoptosis

**FIGURE 8:**
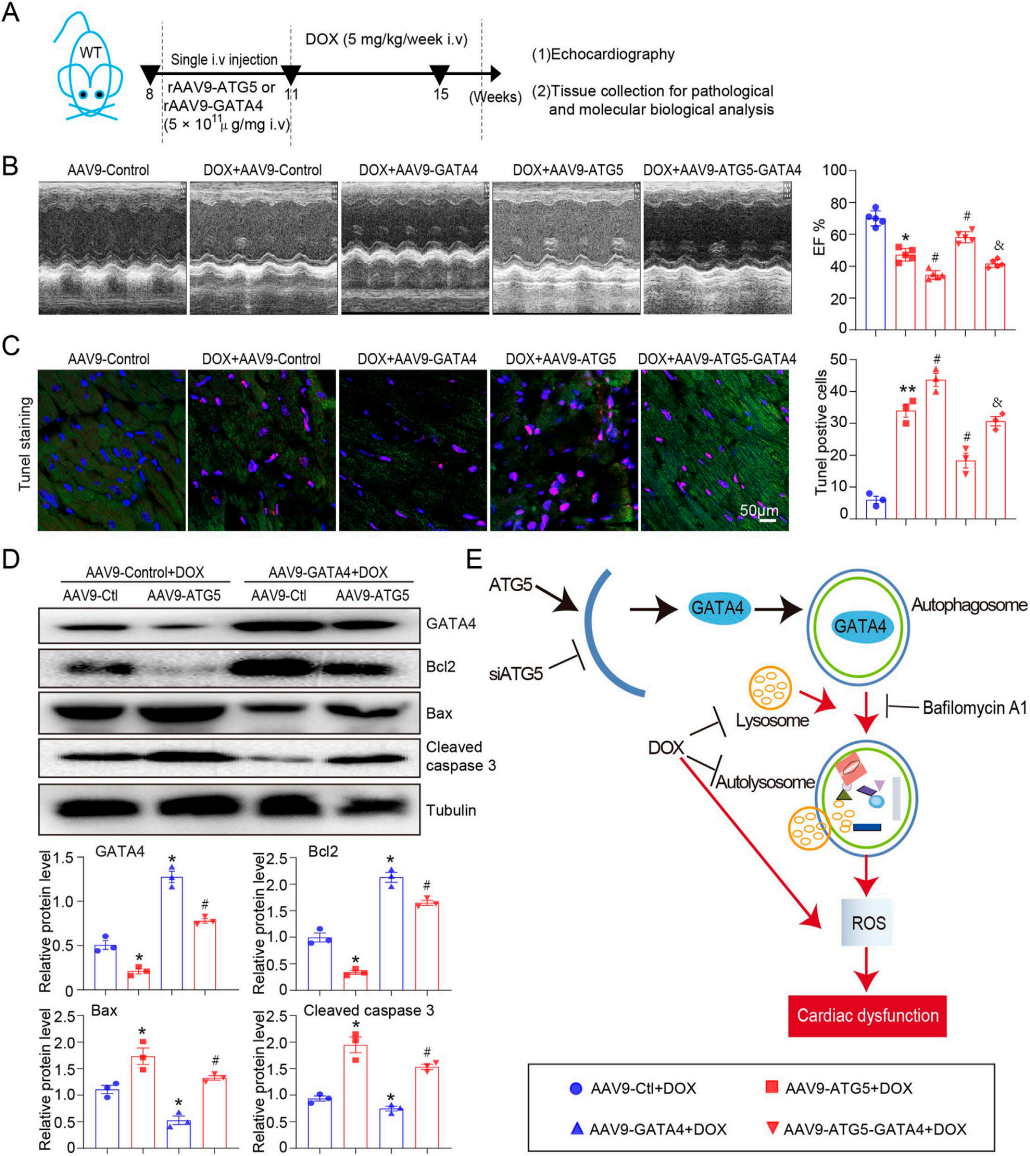
Enforced expression of GATA4 in the heart reversed DOX-induced cardiac dysfunction and apoptosis, aggravated by ATG5. **(A)** Mice were intravenously administered a single rAAV9-ATG5 and rAAV9-GATA4 for 3 weeks, followed by DOX for another 4 weeks. **(B)** Dynamics of ejection fraction (EF)% and fractional shortening (FS)% in mice were examined using a high-frequency echo system (n = 5). **(C)** Representative images of Tunnel staining and the quantitative results revealing the myocardial apoptosis in heart tissues (n = 3). **(D)** Representative Western blotting images and quantitative analysis of GATA4, Bcl2, Bax, and Caspase3 in the heart tissues (n = 3). **(E)** Schematic model: ATG5-mediated autophagy promoted GATA4 degradation and aggravated DOX-induced cardiac dysfunction. Data are presented as the mean ± SEM. Statistical significance was determined using one-way ANOVA with Tukey’s *post hoc* test. *P < 0.05 versus AAV9-Ctl + DOX group; #*P* < 0.05 versus AAV9-ATG5 + DOX group.

To further confirm the molecular mechanism by which ATG5-mediated autophagy promoted GATA4 degradation that aggravated DOX-induced myocardial dysfunction and apoptosis, rAAV9-GATA4 was constructed and transfected into the heart to restore GATA4 level (Figure 8A). Compared with the DOX + rAAV9-Control group, LVEF was significantly decreased in the DOX + rAAV9-ATG5 group, and this effect was significantly reversed by rAAV9-GATA4 (Figure 8B). Moreover, DOX-induced myocardial apoptosis, aggravated by ATG5 overexpression, was reversed by rAAV9-GATA4 (Figure 8C). Moreover, Western blotting results demonstrated that rAAV9-GATA4 successfully restored GATA4, enhanced the protein level of Bcl-2, and reduced the protein levels of Bax and cleaved caspase 3 in DOX-treated hearts (Figure 8D). Moreover, enforced expression of GATA4 significantly inhibited the effects of ATG5 on Bcl-2, Bax, and cleaved caspase 3 protein expression in DOX-treated hearts (Figure 8D).

## Discussion

The present study uncovered the detrimental role of ATG5 in DOX-induced cardiac dysfunction. We provide data to confirm that DOX reduces GATA4 expression and stimulates autophagy initiation (by upregulating *Atg* genes for ATG5), simultaneously impairing the function of available lysosomes and preventing their biogenesis, resulting in autophagosome accumulation and inhibition of flux. Knockdown ATG5 restored GATA4 level and mitigated DOX-induced cardiac dysfunction, fibrosis, and oxidative stress, whereas overexpression ATG5 aggravated these effects (Figure 8E). Therefore, our study highlights ATG5-mediated autophagy as a potential therapeutic target in DOX-induced cardiac dysfunction.

Autophagy is a biological process that promotes the degradation of dysfunctional and long-lived proteins and injured organelles (Deretic and Kroemer, 2022). Autophagy activation was involved in certain cardiovascular diseases, including myocardial ischemia (Chen et al., 2021), pathological cardiac hypertrophy (Zeng et al., 2022), heart failure (Du et al., 2020), and diabetic cardiomyopathy (Bravo-San Pedro et al., 2017; Zhang M. et al., 2021). In the model of DOX-induced cardiotoxicity, LC3-II protein expression was increased in the heart, and this effect was due to inhibition of autophagosome-lysosome fusion (Li et al., 2016). Regulation of key steps responsible for autophagy may exert a protective or detrimental role in DOX-induced cardiotoxicity. For example, inhibition of autophagy by knockout Beclin 1 reduced ROS generation and improved cardiac function in DOX-treated mice (Li et al., 2016). Additionally, transgenic overexpression of Beclin 1 in the heart further increases DOX-induced ROS generation, aggravating cardiac dysfunction (Li et al., 2016). In a DOX-induced cardiotoxicity model of zebrafish, atg7 overexpression improved cardiac function in the late phase but deleterious effects in the early phase of DOX-induced cardiotoxicity (Wang Y. et al., 2021). ATG5 knockdown by rAAV9-ATG5 alleviated DOX-induced mitochondrial ROS emission at acute time points in Sprague Dawley rats (Montalvo et al., 2020). The results indicated that ATG5 knockdown by rAAV9-shRNA-ATG5 in mice for 7 weeks alleviated DOX-induced cardiac dysfunction, cardiac fibrosis, oxidative stress, and myocardial apoptosis (Figures 3B, 4A), and ATG5 overexpression by rAAV9-ATG5 exerted the opposite effects. We hypothesized that the different results of our study and those of others could be attributed to the various animal models employed. In this study, we selected 8–10 weeks of male C57BL/6 mice instead of rats in another study, and the mice were subjected to DOX through intravenous injection instead of intraperitoneal injection. Intraperitoneal administration of DOX may influence the accuracy of experimental results obtained in the heart owing to its side effects, including peritoneal fibrosis, consequent malaise, anorexia, weight loss, and noncardiac death. Decreased food intake-induced starvation results in autophagy (Singh, 2012), and the damaged peritoneum impedes DOX absorption in the ensuing injections. Therefore, an intravenous injection of 5 mg/kg DOX was used in this study. According to a previous study (Li et al., 2016), this dose of DOX had minimal side effects.

Previous studies have investigated the essential role of DOX in cardiomyocytes, but the conclusions were conflicting (Xiao et al., 2019). Autophagy were reported to be increased or reduced in DOX-treated hearts (Xiao et al., 2019), and we speculated the controversial conclusion were attribute to several factors: (1) The animal model were different, most of them used acute, high-dose doxorubicin to establish the cardiac toxic model which do not accurately reflect the clinical scenario of chronic doxorubicin cardiomyopathy. (2) Intraperitoneal administration of DOX resulted in malaise, anorexia, and cachexia. In this study, we established a chronic cardiac toxic model by intravenously injections of DOX at doses employed clinically, and we thought this model could avoid the numerous confounding comorbidities which plague other studies. Meanwhile, the effects of DOX on H9c2 cell was also controversial. Several studies reported DOX increased LC3 puncta and induced autophagy by AKT/mTOR pathway, thus exerted a toxic effect by inducing oxidative stress and apoptosis (Wang et al., 2018; Zhou et al., 2022), but other’s studies suggested that DOX blocks autophagic flux through inhibiting lysosomal acidification (Li et al., 2016) and activating E2F transcription factor 1 (E2F1)/mTOR (Gu et al., 2018). And autophagy activation improved DOX induced oxidative stress and apoptosis. These controversial results at a large extent depend on different DOX dose and observation time. Considering the undefined relationship between autophagy and DOX, we here used mRFP-GFP-LC3 probe revealed an increase in autophagic flux after DOX treatment (Figure 2C). We also found that DOX reduce the co-localization of LC3-II and LAMP1 (Supplementary Figure S1A), suggesting that DOX may increase autophagic flux but inhibited autophagic degradation.

Increased ROS generation is the primary cause of DOX-induced cardiotoxicity (Kong et al., 2022), and supplementation with antioxidants has been demonstrated to alleviate DOX-induced cardiotoxicity (Kong et al., 2022; Cadeddu Dessalvi et al., 2021). Previous studies have demonstrated that inhibition of DOX-induced autophagy is associated with enhanced expression of antioxidant response-related genes in the soleus, including *GPX1, SOD1*, and *SOD2* (Doerr et al., 2020). ATG5 knockdown reduces chemotherapy-induced oxidative stress in osteosarcoma cells (Hollomon et al., 2013). Importantly, SQSTM1/p62 is known to mediate the selective autophagy of GATA4, and GATA4 further increased NF-κB activity that underlies the increased expression of cytokines and reduction of reactive oxygen species (Liu et al., 2021). Consistent with these findings, our results suggested that inhibition of autophagosome formation by rAAV9-siRNA-ATG5 in the heart restored GATA4 level and mitigated DOX-induced oxidative stress and myocardial apoptosis (Figures 4, 7). These findings indicate that autophagy occurs upstream of DOX-induced oxidative stress, and ATG5-mediated GATA4 degradation may partly contribute to the oxidative stress in DOX-treated heart.

The role of autophagy in regulating cardiac fibrosis has been well described. Recent studies suggested a pro-fibrotic role for autophagy (Liang et al., 2022). Advanced glycation end products (AGEs) and receptor for advanced glycation end products (RAGE) axis is involved in myocardial fibrosis in heart failure through cardiac fibroblasts activation induced by autophagy (Liang et al., 2022). Knockout RAGE decreased endothelial-to-mesenchymal transition accompanied by decreased expression of autophagy-related proteins (LC3BII/I and Beclin 1), and alleviated cardiac fibrosis and improved cardiac function in TAC mice (Zhang L. et al., 2021). However, other’s studies suggested that autophagy induction promoted cardiac fibrosis (Liu et al., 2016), and autophagy inhibition alleviated cardiac fibrosis (Gupta et al., 2016). TGF-β1 simultaneously induces autophagy and fibrosis in human cardiac myofibroblasts and pharmacological inhibition of autophagy is associated with a parallel reduction in TGF-β1-induced fibrosis (Ghavami et al., 2015). In our research, we found the increased protein expression of TGF-β and p-smad2/3 caused by DOX was further increased by rAAV9-ATG5. Thus, our study indicated that enhancing autophagy by overexpressing ATG5 aggravated DOX induced cardiac fibrosis via TGF-β/p-Smad signal pathway.

GATA4 protein expression was reduced in DOX-treated hearts, and enforced expression of GATA4 in the heart could upregulate anti-apoptotic genes *Bcl-2 and Bcl-xL*, further alleviating DOX-induced myocardial apoptosis and cardiac dysfunction (Aries et al., 2004). Corresponding to this finding, we observed that DOX increased Bax and Caspase-3 expression and inhibited induced myocardial apoptosis in ATG5 knockdown hearts (Figure 4), whereas these effects were aggravated in ATG5 overexpressing hearts (Figure 6). GATA4 can be regulated by autophagic degradation (Song et al., 2021; Chen et al., 2022), whereas autophagy can be regulated by GATA4 (Kobayashi et al., 2010). GATA4 can be ubiquitinated and then recognised by p62, is translocated to autophagosomes to form autophagolysosomes and degraded (Chen et al., 2022). Blocking GATA4 autophagic degradation promoted cardiac hypertrophy (Song et al., 2021). On the other hand, some researches believe that the ability of GATA4 to inhibit autophagy is mediated at least partly by its downstream effector Bcl2 (Kobayashi et al., 2010). The anti-autophagic effect of Bcl2 is reported to be achieved through its interaction with Beclin 1 (Atg6), a protein essential for autophagy initiation. Although overexpression of GATA4 did not have effect on ATG genes at base line, it repressed DOX-induced expression of ATG genes (Kobayashi et al., 2010). Importantly, GATA4 gene silencing resulted in a dramatic upregulation of ATG5, ATG7, ATG1 and Beclin 1 (Kobayashi et al., 2010). In this study, GATA4 was reduced in cardiomyocytes treated with autophagy inhibitors 3-MA, CQ, and siATG5, as well as in cardiac tissues of mice after intravenous injection of AAV9-shATG5 (Figure 7). Conversely, GATA4 expression was increased in cardiomyocytes and cardiac tissues after autophagy induction by RAPA, adenovirus, or AAV9 expressing ATG5 (Figure 7). These findings suggest that ATG5-mediated autophagy regulates GATA4 degradation, thereby further regulating cardiac function, oxidative stress and apoptosis in DOX-treated hearts.

Autophagy can be divided into microautophagy, macroautophagy, and chaperone mediated autophagy (CMA) (Massey et al., 2006). CMA is the only autophagic pathway that allows selective degradation of soluble proteins in lysosomes (Massey et al., 2006). In contrast to the other mammalian forms of autophagy, CMA does not require vesicle formation or major changes in the lysosomal membrane (Massey et al., 2006). The substrate proteins are targeted to the lysosomal membrane by recognition of a targeting motif, by a chaperone complex, consisting of hsc70 and its co-chaperones (Massey et al., 2006). It should be emphasized that p62-mediated lysosomal degradation of GATA4 was not HSC70-mediated CMA (Chen et al., 2022). Our data revealed that ATG5-mediated autophagic degradation of GATA4 is mediated by p62 (Figure 7), so we speculated that ATG5-mediated autophagic degradation of GATA4 in this study was not CMA.

It is widely recognized that when autophagy is inhibited, p62 accumulates, while when autophagy is induced, p62 quantities decrease (Jiang and Mizushima, 2015). It has been reported that the protein level of p62 was reduced in DOX-treated H9c2 cells and neonatal rat ventricular myocytes, which indicated the autophagic flux inhibition (Ye et al., 2024). While other study revealed increased p62 levels in the hearts of rat and mouse models post-Dox (1–6 days) (Koleini and Kardami, 2017). We speculated that the controversial results were attributed to the differences in the mode of DOX administration, dosage and duration of action. Interestingly, recent study revealed that p62 expression level is inversely associated with ATG5 (Klionsky et al., 2021; Xu et al., 2020). Conversely, cardiac-specific knockdown of ATG5 was associated with a significant increase in p62 expression (Wang F. et al., 2021). In our research, we found a increased protein level of p62 in DOX treated H9c2 cells and hearts (Figures 1E, 2A), and overexpression of ATG5 reduced the fluorescence intensity of p62 (Figure 7F), these results were consistent with previous research (Xu et al., 2020; Wang F. et al., 2021).

In conclusion, our research demonstrated that ATG5 knockdown reduced autophagosome formation, restored GATA4 expression by inhibiting its degradation, and alleviated DOX-induced cardiac dysfunction, fibrosis, oxidative stress, and apoptosis. These findings suggest that ATG5-mediated autophagy may be a potential therapeutic target for treating DOX cardiotoxicity.

## Data Availability

The original contributions presented in the study are included in the article/Supplementary Material, further inquiries can be directed to the corresponding authors.

## References

[B1] Aries A. Paradis P. Lefebvre C. Schwartz R. J. Nemer M. (2004). Essential role of GATA-4 in cell survival and drug-induced cardiotoxicity. Proc. Natl. Acad. Sci. U. S. A. 101, 6975–6980. 10.1073/pnas.0401833101 15100413 PMC406451

[B2] Aries A. Whitcomb J. Shao W. Komati H. Saleh M. Nemer M. (2014). Caspase-1 cleavage of transcription factor GATA4 and regulation of cardiac cell fate. Cell Death Dis. 5, e1566. 10.1038/cddis.2014.524 25501827 PMC4649840

[B3] Bravo-San Pedro J. M. Kroemer G. Galluzzi L. (2017). Autophagy and mitophagy in cardiovascular disease. Circ. Res. 120, 1812–1824. 10.1161/CIRCRESAHA.117.311082 28546358

[B4] Cadeddu Dessalvi C. Deidda M. Noto A. Madeddu C. Cugusi L. Santoro C. (2021). Antioxidant approach as a cardioprotective strategy in chemotherapy-induced cardiotoxicity. Antioxid. Redox Signal 34, 572–588. 10.1089/ars.2020.8055 32151144

[B5] Chai Q. Zhang W. Gao L. Yang Y. Miao M. Liu D. (2023). The action and mechanism of trehalose on GATA4 autophagy degradation and ventricular remodeling. Discov. Med. 35, 394–404. 10.24976/Discov.Med.202335176.40 37272106

[B6] Chen H. Zhou J. Chen H. Liang J. Xie C. Gu X. (2022). Bmi-1-RING1B prevents GATA4-dependent senescence-associated pathological cardiac hypertrophy by promoting autophagic degradation of GATA4. Clin. Transl. Med. 12, e574. 10.1002/ctm2.574 35390228 PMC8989148

[B7] Chen H. Y. Xiao Z. Z. Ling X. Xu R. N. Zhu P. Zheng S. Y. (2021). ELAVL1 is transcriptionally activated by FOXC1 and promotes ferroptosis in myocardial ischemia/reperfusion injury by regulating autophagy. Mol. Med. 27, 14. 10.1186/s10020-021-00271-w 33568052 PMC7874472

[B8] Cicchini M. Karantza V. Xia B. (2015). Molecular pathways: autophagy in cancer--a matter of timing and context. Clin. Cancer Res. 21, 498–504. 10.1158/1078-0432.ccr-13-2438 25165101 PMC4315744

[B9] Deretic V. Kroemer G. (2022). Autophagy in metabolism and quality control: opposing, complementary or interlinked functions? Autophagy 18, 283–292. 10.1080/15548627.2021.1933742 34036900 PMC8942406

[B10] Doerr V. Montalvo R. N. Kwon O. S. Talbert E. E. Hain B. A. Houston F. E. (2020). Prevention of doxorubicin-induced autophagy attenuates oxidative stress and skeletal muscle dysfunction. Antioxidants (Basel) 9, 263. 10.3390/antiox9030263 32210013 PMC7139604

[B11] Du J. Liu Y. Fu J. (2020). Autophagy and heart failure. Adv. Exp. Med. Biol. 1207, 223–227. 10.1007/978-981-15-4272-5_16 32671751

[B12] Ghavami S. Cunnington R. H. Gupta S. Yeganeh B. Filomeno K. L. Freed D. H. (2015). Autophagy is a regulator of TGF-β1-induced fibrogenesis in primary human atrial myofibroblasts. Cell Death Dis. 6, e1696. 10.1038/cddis.2015.36 25789971 PMC4385916

[B13] Gu J. Fan Y. Q. Zhang H. L. Pan J. A. Yu J. Y. Zhang J. F. (2018). Resveratrol suppresses doxorubicin-induced cardiotoxicity by disrupting E2F1 mediated autophagy inhibition and apoptosis promotion. Biochem. Pharmacol. 150, 202–213. 10.1016/j.bcp.2018.02.025 29475062

[B14] Gupta S. S. Zeglinski M. R. Rattan S. G. Landry N. M. Ghavami S. Wigle J. T. (2016). Inhibition of autophagy inhibits the conversion of cardiac fibroblasts to cardiac myofibroblasts. Oncotarget 7, 78516–78531. 10.18632/oncotarget.12392 27705938 PMC5346657

[B15] Hollomon M. G. Gordon N. Santiago-O'Farrill J. M. Kleinerman E. S. (2013). Knockdown of autophagy-related protein 5, ATG5, decreases oxidative stress and has an opposing effect on camptothecin-induced cytotoxicity in osteosarcoma cells. BMC Cancer 13, 500. 10.1186/1471-2407-13-500 24160177 PMC3924338

[B16] Iriondo M. N. Etxaniz A. Varela Y. R. Ballesteros U. Lazaro M. Valle M. (2023). Effect of ATG12-ATG5-ATG16L1 autophagy E3-like complex on the ability of LC3/GABARAP proteins to induce vesicle tethering and fusion. Cell Mol. Life Sci. 80, 56. 10.1007/s00018-023-04704-z 36729310 PMC9894987

[B17] Jiang P. Mizushima N. (2015). LC3- and p62-based biochemical methods for the analysis of autophagy progression in mammalian cells. Methods 75, 13–18. 10.1016/j.ymeth.2014.11.021 25484342

[B18] Klionsky D. J. Abdel-Aziz A. K. Abdelfatah S. Abdellatif M. Abdoli A. Abel S. (2021). Guidelines for the use and interpretation of assays for monitoring autophagy (4th edition)^1^ . Autophagy 17, 1–382. 10.1080/15548627.2020.1797280 33634751 PMC7996087

[B19] Kobayashi S. Lackey T. Huang Y. Bisping E. Pu W. T. Boxer L. M. (2006). Transcription factor gata4 regulates cardiac BCL2 gene expression *in vitro* and *in vivo* . FASEB J. 20, 800–802. 10.1096/fj.05-5426fje 16469847

[B20] Kobayashi S. Volden P. Timm D. Mao K. Xu X. Liang Q. (2010). Transcription factor GATA4 inhibits doxorubicin-induced autophagy and cardiomyocyte death. J. Biol. Chem. 285, 793–804. 10.1074/jbc.M109.070037 19901028 PMC2804228

[B21] Koleini N. Kardami E. (2017). Autophagy and mitophagy in the context of doxorubicin-induced cardiotoxicity. Oncotarget 8, 46663–46680. 10.18632/oncotarget.16944 28445146 PMC5542301

[B22] Kong C. Y. Guo Z. Song P. Zhang X. Yuan Y. P. Teng T. (2022). Underlying the mechanisms of doxorubicin-induced acute cardiotoxicity: oxidative stress and cell death. Int. J. Biol. Sci. 18, 760–770. 10.7150/ijbs.65258 35002523 PMC8741835

[B23] Li D. L. Wang Z. V. Ding G. Tan W. Luo X. Criollo A. (2016). Doxorubicin blocks cardiomyocyte autophagic flux by inhibiting lysosome acidification. Circulation 133, 1668–1687. 10.1161/CIRCULATIONAHA.115.017443 26984939 PMC4856587

[B24] Liang B. Zhou Z. Yang Z. Liu J. Zhang L. He J. (2022). AGEs-RAGE axis mediates myocardial fibrosis via activation of cardiac fibroblasts induced by autophagy in heart failure. Exp. Physiol. 107, 879–891. 10.1113/EP090042 35598104

[B25] Liang Q. Molkentin J. D. (2002). Divergent signaling pathways converge on GATA4 to regulate cardiac hypertrophic gene expression. J. Mol. Cell Cardiol. 34, 611–616. 10.1006/jmcc.2002.2011 12054848

[B26] Liao Z. Q. Jiang Y. N. Su Z. L. Bi H. L. Li J. T. Li C. L. (2021). Rutaecarpine inhibits doxorubicin-induced oxidative stress and apoptosis by activating AKT signaling pathway. Front. Cardiovasc Med. 8, 809689. 10.3389/fcvm.2021.809689 35071368 PMC8766983

[B27] Liu S. Chen S. Li M. Zhang B. Shen P. Liu P. (2016). Autophagy activation attenuates angiotensin II-induced cardiac fibrosis. Arch. Biochem. Biophys. 590, 37–47. 10.1016/j.abb.2015.11.001 26562437

[B28] Liu X. Zhao M. Sun X. Meng Z. Bai X. Gong Y. (2021). Autophagic flux unleashes GATA4-NF-*κ*b Axis to promote antioxidant defense-dependent survival of colorectal cancer cells under chronic acidosis. Oxid. Med. Cell Longev. 2021, 8189485. 10.1155/2021/8189485 34987705 PMC8720590

[B29] Ljubojevic-Holzer S. Kraler S. Djalinac N. Abdellatif M. Voglhuber J. Schipke J. (2022). Loss of autophagy protein ATG5 impairs cardiac capacity in mice and humans through diminishing mitochondrial abundance and disrupting Ca2+ cycling. Cardiovasc Res. 118, 1492–1505. 10.1093/cvr/cvab112 33752242 PMC9074988

[B30] Massey A. C. Zhang C. Cuervo A. M. (2006). Chaperone-mediated autophagy in aging and disease. Curr. Top. Dev. Biol. 73, 205–235. 10.1016/S0070-2153(05)73007-6 16782460

[B31] Mizuta Y. Tokuda K. Guo J. Zhang S. Narahara S. Kawano T. (2020). Sodium thiosulfate prevents doxorubicin-induced DNA damage and apoptosis in cardiomyocytes in mice. Life Sci. 257, 118074. 10.1016/j.lfs.2020.118074 32673667

[B32] Montalvo R. N. Doerr V. Kwon O. S. Talbert E. E. Yoo J. K. Hwang M. H. (2020). Protection against doxorubicin-induced cardiac dysfunction is not maintained following prolonged autophagy inhibition. Int. J. Mol. Sci. 21, 8105. 10.3390/ijms21218105 33143122 PMC7662380

[B33] Oka T. Maillet M. Watt A. J. Schwartz R. J. Aronow B. J. Duncan S. A. (2006). Cardiac-specific deletion of Gata4 reveals its requirement for hypertrophy, compensation, and myocyte viability. Circ. Res. 98, 837–845. 10.1161/01.RES.0000215985.18538.c4 16514068

[B34] Rawat P. S. Jaiswal A. Khurana A. Bhatti J. S. Navik U. (2021). Doxorubicin-induced cardiotoxicity: an update on the molecular mechanism and novel therapeutic strategies for effective management. Biomed. Pharmacother. 139, 111708. 10.1016/j.biopha.2021.111708 34243633

[B35] Singh R. (2012). Autophagy in the control of food intake. Adipocyte 1, 75–79. 10.4161/adip.18966 23700515 PMC3609083

[B36] Song R. Lei H. Feng L. Cheng W. Li Y. Yao L. L. (2021). TFEB insufficiency promotes cardiac hypertrophy by blocking autophagic degradation of GATA4. J. Biol. Chem. 297, 101189. 10.1016/j.jbc.2021.101189 34517007 PMC8498468

[B37] Taneike M. Yamaguchi O. Nakai A. Hikoso S. Takeda T. Mizote I. (2010). Inhibition of autophagy in the heart induces age-related cardiomyopathy. Autophagy 6, 600–606. 10.4161/auto.6.5.11947 20431347

[B38] Wang F. He Q. Gao Z. Redington A. N. (2021b). Atg5 knockdown induces age-dependent cardiomyopathy which can be rescued by repeated remote ischemic conditioning. Basic Res. Cardiol. 116, 47. 10.1007/s00395-021-00888-2 34319513 PMC8316897

[B39] Wang H. Wang H. Liang E. Y. Zhou L. X. Dong Z. L. Liang P. (2018). Thrombopoietin protects H9C2 cells from excessive autophagy and apoptosis in doxorubicin-induced cardiotoxicity. Oncol. Lett. 15, 839–848. 10.3892/ol.2017.7410 29403560 PMC5780751

[B40] Wang Y. Lu X. Wang X. Qiu Q. Zhu P. Ma L. (2021a). atg7-Based autophagy activation reverses doxorubicin-induced cardiotoxicity. Circ. Res. 129, e166–e182. 10.1161/CIRCRESAHA.121.319104 34384247 PMC8484060

[B41] Wu L. Wang L. Du Y. Zhang Y. Ren J. (2023). Mitochondrial quality control mechanisms as therapeutic targets in doxorubicin-induced cardiotoxicity. Trends Pharmacol. Sci. 44, 34–49. 10.1016/j.tips.2022.10.003 36396497

[B42] Xiao B. Hong L. Cai X. Mei S. Zhang P. Shao L. (2019). The true colors of autophagy in doxorubicin-induced cardiotoxicity. Oncol. Lett. 18, 2165–2172. 10.3892/ol.2019.10576 31452719 PMC6676529

[B43] Xu C. N. Kong L. H. Ding P. Liu Y. Fan Z. G. Gao E. H. (2020). Melatonin ameliorates pressure overload-induced cardiac hypertrophy by attenuating Atg5-dependent autophagy and activating the Akt/mTOR pathway. Biochim. Biophys. Acta Mol. Basis Dis. 1866, 165848. 10.1016/j.bbadis.2020.165848 32473999

[B44] Yamamoto H. Zhang S. Mizushima N. (2023). Autophagy genes in biology and disease. Nat. Rev. Genet. 24, 382–400. 10.1038/s41576-022-00562-w 36635405 PMC9838376

[B45] Ye C. Yan C. Bian S. J. Li X. R. Li Y. Wang K. X. (2024). Momordica charantia L.-derived exosome-like nanovesicles stabilize p62 expression to ameliorate doxorubicin cardiotoxicity. J. Nanobiotechnology 22, 464. 10.1186/s12951-024-02705-z 39095755 PMC11297753

[B46] Yuan L. Ji H. G. Yan X. J. Liu M. Ding Y. H. Chen X. H. (2023). Dioscin ameliorates doxorubicin-induced heart failure via inhibiting autophagy and apoptosis by controlling the PDK1-mediated Akt/mTOR signaling pathway. Kaohsiung J. Med. Sci. 39, 1022–1029. 10.1002/kjm2.12740 37578093 PMC11895924

[B47] Zeng Y. Ren W. Q. Wen A. Z. Zhang W. Fan F. Y. Chen O. Y. (2022). Autophagy and pressure overload-induced cardiac hypertrophy. J. Asian Nat. Prod. Res. 24, 1101–1108. 10.1080/10286020.2021.2024810 35043747

[B48] Zhang L. He J. Wang J. Liu J. Chen Z. Deng B. (2021b). Knockout RAGE alleviates cardiac fibrosis through repressing endothelial-to-mesenchymal transition (EndMT) mediated by autophagy. Cell Death Dis. 12, 470. 10.1038/s41419-021-03750-4 33976108 PMC8113558

[B49] Zhang M. Sui W. Xing Y. Cheng J. Cheng C. Xue F. (2021a). Angiotensin IV attenuates diabetic cardiomyopathy via suppressing FoxO1-induced excessive autophagy, apoptosis and fibrosis. Theranostics 11, 8624–8639. 10.7150/thno.48561 34522203 PMC8419053

[B50] Zhou L. Han Y. Yang Q. Xin B. Chi M. Huo Y. (2022). Scutellarin attenuates doxorubicin-induced oxidative stress, DNA damage, mitochondrial dysfunction, apoptosis and autophagy in H9c2 cells, cardiac fibroblasts and HUVECs. Toxicol Vitro 82, 105366. 10.1016/j.tiv.2022.105366 35470029

