# Supplementary figures and images for: Knockdown ATG5 gene by rAAV9 alleviates doxorubicin-induced cardiac toxicity by inhibiting GATA4 autophagic degradation
Source: Front Pharmacol. 2025 Jan 27;15:1496380. doi: 10.3389/fphar.2024.1496380 (PMC11808915; doi:10.3389/fphar.2024.1496380)

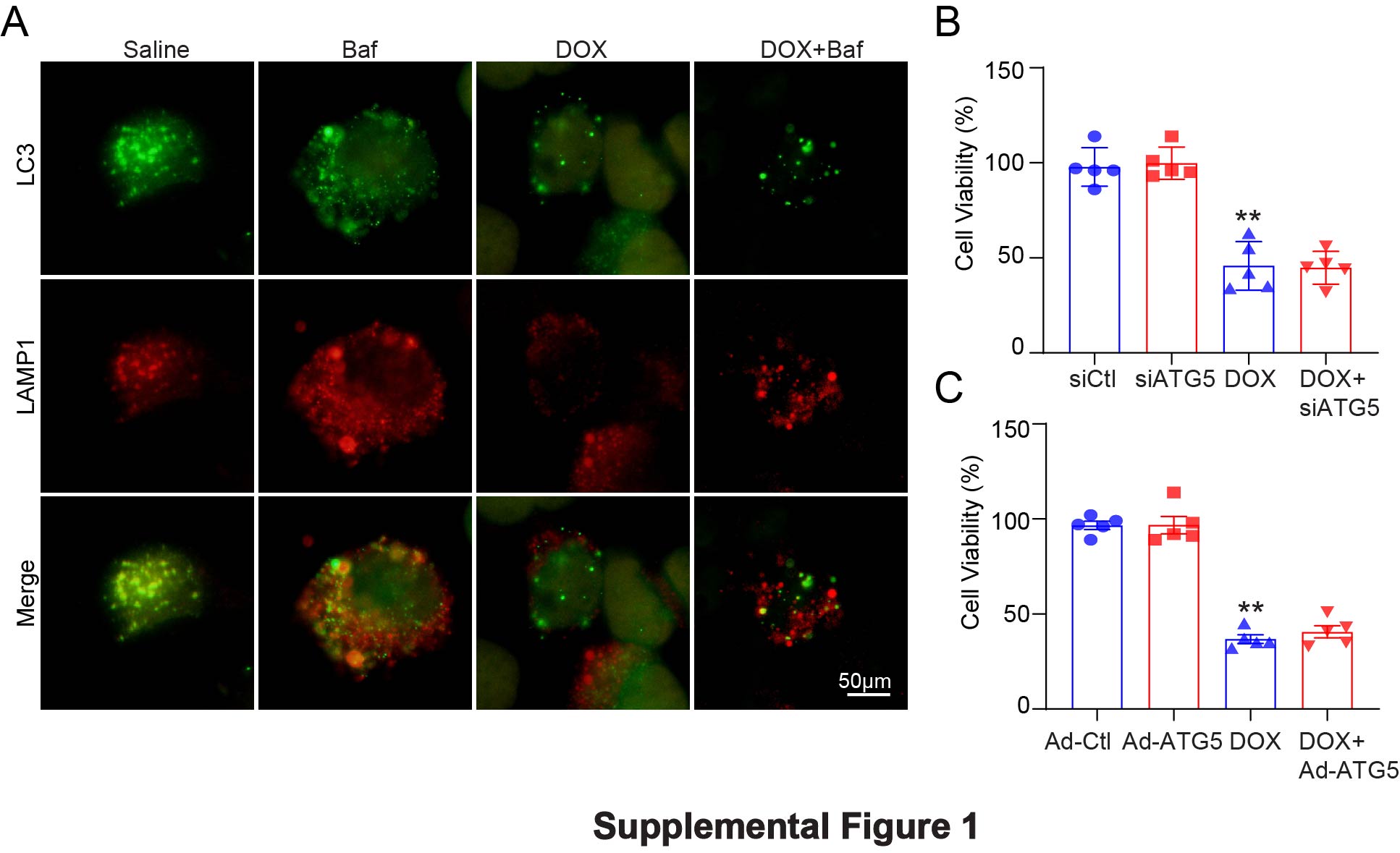

Supplement: Supplementary file 1 [file Image1.jpeg]
